# Tumor Extrinsic Factors Mediate Primary T-DM1 Resistance in HER2-Positive Breast Cancer Cells

**DOI:** 10.3390/cancers13102331

**Published:** 2021-05-12

**Authors:** Yukinori Endo, Wen Jin Wu

**Affiliations:** Division of Biotechnology Review and Research 1, Office of Biotechnology Products, Office of Pharmaceutical Quality, Center for Drug Evaluation and Research, U.S. Food and Drug Administration (FDA), Silver Spring, MD 20993, USA; Yukinori.Endo@fda.hhs.gov

**Keywords:** HER2, breast cancer, tumor microenvironment, EGFR, antibody-drug conjugate (ADC), T-DM1, primary drug resistance, Matrigel, extrinsic factor

## Abstract

**Simple Summary:**

In this investigation, we employed an unconventional approach to explore the mechanisms of the primary resistance of human epidermal growth factor 2 (HER2)-positive breast cancer cells to ado-trastuzumab emtansine (also known as T-DM1). Specifically, we used Matrigel matrix as a model of the tumor microenvironment and examined its effect on the sensitivity of HER2-positive breast cancer cells to T-DM1. We found that epidermal growth factor receptor (EGFR) is activated in HER2-positive, T-DM1-sensitive JIMT1 and SKBR-3 cells on the Matrigel matrix. This leads to phosphorylation and degradation of HER2 in these cells, resulting in the loss of or reduced sensitivity to T-DM1. The discovery of extrinsic factors contributing to the primary resistance of HER2-positive breast cancer cells to T-DM1 provides an opportunity to develop a novel therapeutic strategy to overcome T-DM1 resistance.

**Abstract:**

To explore if the tumor microenvironment contributes to the primary resistance of HER2-positive breast cancer cells to T-DM1, we examined whether Matrigel, a basement membrane matrix that provides a three-dimensional (3D) cell culture condition, caused the primary resistance of HER2-positive, T-DM1-sensitive breast cancer cells (JIMT1 and SKBR-3 cells) to T-DM1. This is different from the conventional approach such that the cells are exposed with escalated doses of drug to establish a drug-resistant cell line. We found that these cells were able to grow and form spheroids on the Matrigel in the presence of T-DM1. We further explored the molecular mechanisms that enables these cells to be primarily resistant to T-DM1 and found that EGFR was activated in the spheroids, leading to an increased HER2 tyrosine phosphorylation. This in turn enhances cell growth signaling downstream of EGFR/HER2 in the spheroids. HER2 tyrosine phosphorylation promotes receptor internalization and degradation in the spheroids, which limits T-DM1 access to HER2 on the cell surface of spheroids. Blocking EGFR activity by erlotinib reduces HER2 tyrosine phosphorylation and enhances HER2 cell surface expression. This enables T-DM1 to gain access to HER2 on the cell surface, resumes cell sensitivity to T-DM1, and exhibits synergistic activity with T-DM1 to inhibit the formation of spheroids on Matrigel. The discovery described in this manuscript reveals a novel approach to investigate the primary resistance of HER2-positive breast cancer cells and provides an opportunity to develop a therapeutic strategy to overcome primary resistance to T-DM1 by combing T-DM1 therapy with kinase inhibitors of EGFR.

## 1. Introduction

Despite the promising clinical outcome of targeted therapy for the treatment of cancers, drug resistance is still a major hurdle at the clinic and limits its success [1,2]. Two major types of drug resistance are known as primary (or intrinsic) resistance and acquired (or secondary) resistance. Primary resistance occurs when cancer cells contain some inherent characteristics that prevent them from responding to initial drug treatments. Those cancer patients do not respond to initial therapies [3]. In contrast, acquired resistance refers to cancer cells that develop resistance to initial therapies over time [3]. Both tumor cell-intrinsic (e.g., genetic change in cancer genomes) and -extrinsic (e.g., non-genetic, epigenetic changes such as epithelial-mesenchymal transition) factors are involved in mechanisms of both primary and secondary resistance [3,4]. Tumor microenvironment, including surrounding fibroblasts and stromal cells, growth factors and extracellular matrix (ECM) proteins, is one of the important tumor cell-extrinsic factors [4]. However, it remains largely unclear how the tumor microenvironment contributes to the development of resistance toward specific targeted therapies.

Antibody-drug conjugates (ADCs) typically consist of three structural components: monoclonal antibody (mAbs), linker and payload conjugated to the antibody by the linker [5,6,7]. Ado-trastuzumab emtansine (also known as T-DM1) is an FDA-approved ADC composed of a monoclonal antibody, trastuzumab, specifically targeting HER2 and an anti-mitotic agent, DM1, conjugated to trastuzumab by a thioether linker [8]. Upon binding HER2 on the cell surface of target cells via trastuzumab, T-DM1 is internalized into the cells as a complex with HER2 and undergoes lysosomal degradation to release the active form of DM1 (designated as Lys-MCC-DM1) into the cytoplasm [9,10]. Lys-MCC-DM1 in turn targets microtubules and blocks microtubule polymerization in the target cells where it induces cell apoptosis [9,10].

The primary resistance to T-DM1 was observed in clinical studies for HER2-positive breast cancer patients. In a phase II study (TDM4558g) 29 out of 112 patients (25.9%) achieved an objective response, 55 (49%) had stable disease, and 22 (20%) patients had disease progression with single-agent T-DM1, whereas in the EMILIA trial, 169 out of 397 (42.6%) patients treated with T-DM1 had an objective response [11]. These studies suggest that many patients experience the primary resistance to T-DM1. While several T-DM1 resistance mechanisms have been proposed based on preclinical studies [12,13], the majority of these studies used a conventional approach to establish T-DM1-resistant breast cancer cells by chronically exposing cells to escalating doses of T-DM1 in traditional cell culture dishes. The mechanisms elucidated from these studies demonstrate how cancer cells adapt cell culture environment and acquire resistant to T-DM1 [11,13]. It, however, remains unclear if tumor cell extrinsic factors play an important role in the primary resistance of HER2-positive and T-DM1 sensitive breast cancer cells to T-DM1.

In this study we used a basement membrane Matrigel matrix as a model of the tumor microenvironment to investigate its effects on the sensitivity of HER2-positive breast cancer cells (JIMT1 and SKBR-3 cells) to T-DM1. We found that the Matrigel not only caused cell morphological changes but also activated EGFR, which further activated HER2 and downstream EGFR/HER2 signaling pathways, which promoted cell growth and spheroid formation. We observed that T-DM1 accessibility to cell surface HER2 was lost when HER2-positive breast cancer cells were cultured on the Matrigel matrix. The loss of T-DM1 accessibility to cell surface HER2 was restored by erlotinib, a tyrosine kinase inhibitor of EGFR. Our study provides evidence that the tumor microenvironment alters the target expression of HER2-positive breast cancer cells to render these cells primarily resistant to T-DM1.

## 2. Results

### 2.1. Spheroid Formation of the Parental JIMT1 Cells on Matrigel in the Presence of T-DM1

**Figure 1:**
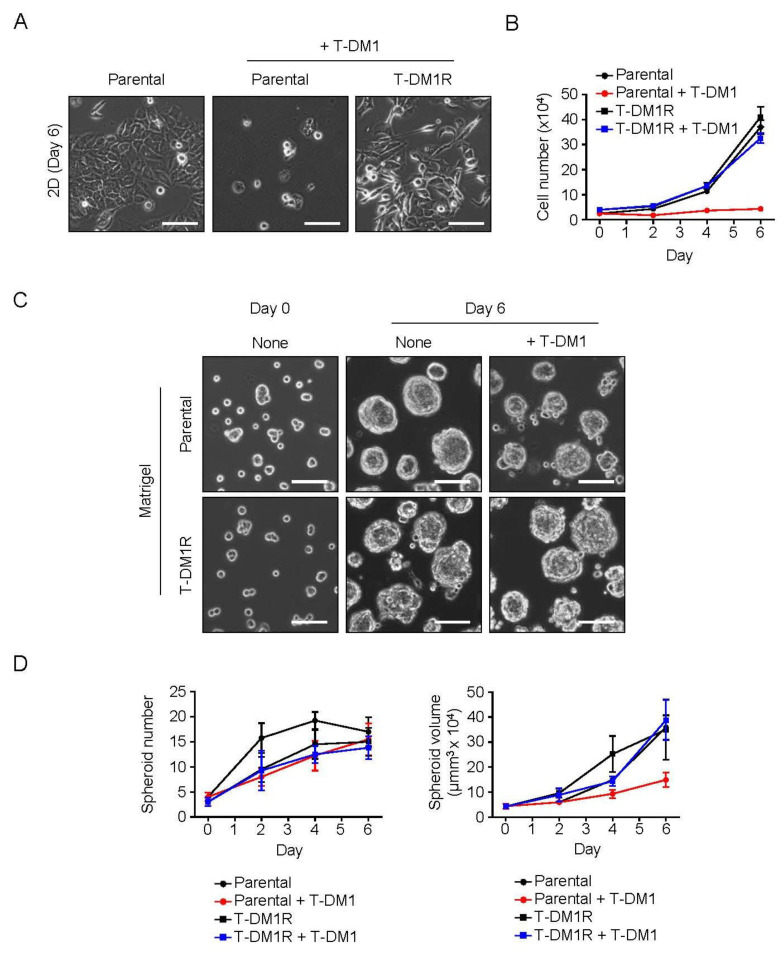
HER2-positive, T-DM1-sensitive breast cancer cells (JIMT1 cells) can grow and form spheroids on the Matrigel matrix in the presence of T-DM1. (**A**) Bright field images of parental JIMT1 cells (indicated as Parental), JIMT1-derived T-DM1-resistant cells (designated as T-DM1R) cultured for 6 days in the absence or presence of 4 µg/mL T-DM1 on the 2D plastic dishes. Scale bar: 100 µm. (**B**) Cell growth profiles of parental and T-DM1R cells either treated with 4 µg/mL T-DM1 or left untreated. (**C**) Bright field images of parental and T-DM1R cells cultured in the absence or presence of 4 µg/mL T-DM1 on Matrigel. Scale bar: 100 µm. (**D**) Spheroid (radius ≥20 µm) growth profiles of parental and T-DM1R cells either treated with 4 µg/mL T-DM1 or left untreated. Spheroid number per image (area of 0.325 mm^2^) on the left panel, spheroid volume on the right panel.

Parental JIMT1 cells were derived from a trastuzumab-resistant, HER2-positive breast cancer patient [14]. In cell culture, these cells do not respond to trastuzumab [15]. As shown in Figure 1A,B, however, parental JIMT1 cells are sensitive to T-DM1 at a dose of 4 µg/mL in conventional two-dimensional (2D) cell culture dishes. Similar results were reported in our previous published paper [12]. We generated T-DM1-resistant JIMT1 cells (T-DM1R) via chronically exposing cells to escalating doses of T-DM1 [12]. To examine whether extrinsic factors or tumor microenvironment can affect the sensitivity of parental JIMT1 cells to T-DM1, the cells were cultured on a Matrigel matrix in the absence or presence of T-DM1 at 4 µg/mL. Matrigel is a basement membrane matrix extracted from the Engelbreth-Holm-Swarm (EHS) mouse sarcoma, and consists of laminin (a major component), collagen IV, heparan sulfate proteoglycans, entactin/nidogen, and a number of growth factors [16,17,18,19]. The Matrigel matrix is widely used as a model of tumor microenvironment [20]. As shown in Figure 1C, both parental JIMT1 and T-DM1R cells displayed different cell morphologies compared to cells cultured on traditional 2D cell culture dishes (compare Figure 1A with Figure 1C). Under the Matrigel system, both parental JIMT1 and T-DM1R cells did not spread but were capable of growing and forming spheroids on the Matrigel matrix (Figure 1C). Surprisingly, both parental JIMT1 cells and T-DM1R cells grew and formed spheroids with similar numbers in the presence of 4 µg/mL T-DM1 on the Matrigel matrix, except that the volumes of parental spheroids were slightly smaller than that of T-DM1R cells (Figure 1C,D). These results indicate that tumor cell-extrinsic factors are able to alter the sensitivity of parental JIMT1 cells to T-DM1 and that the parental JIMT1 cell are primarily resistant to T-DM1 when these cells are cultured on the Matrigel matrix.

### 2.2. EGFR Activation and HER2 Phosphorylation of JIMT1 Cell Spheroids on Matrigel

**Figure 2:**
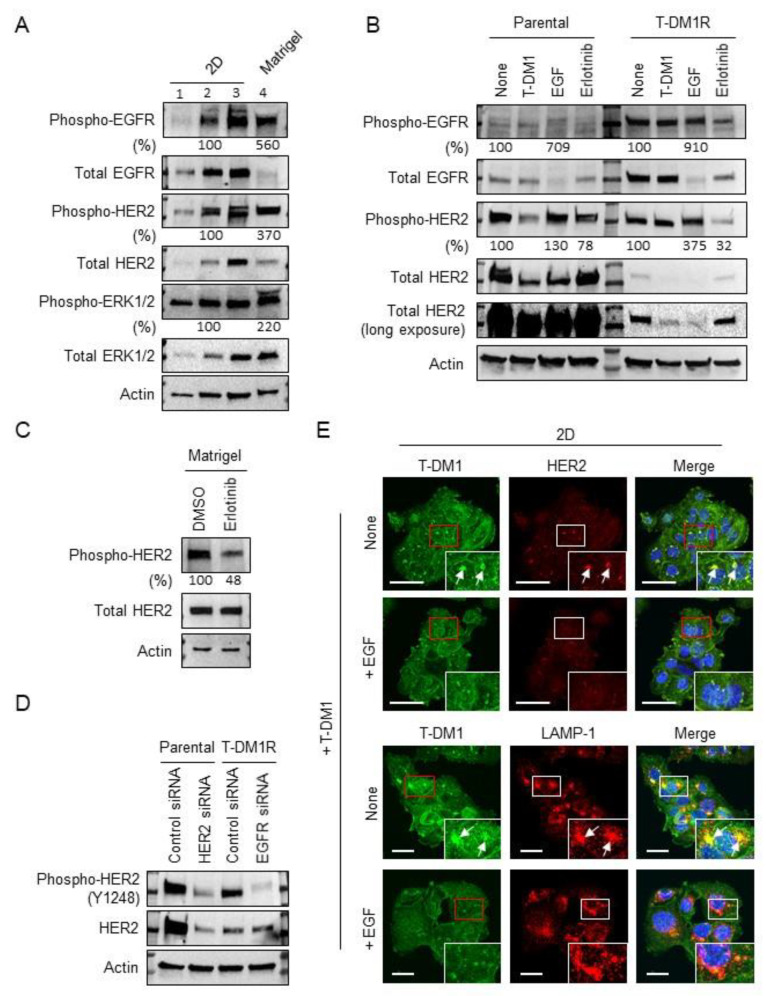
EGFR activation followed by HER2 phosphorylation blocks T-DM1 accessibility to cell surface HER2 in JIMT1 cells grown on a Matrigel matrix and on 2D culture in the presence of EGF. (**A**) The levels of phosphorylated EGFR (Y1045), total EGFR, phosphorylated HER2 (Y1248), total HER2, phosphorylated ERK1/2 and total ERK1/2 expression were evaluated by Western blot in whole-cell lysate (WCL) of parental JIMT1 cells cultured either on 2D (lanes 1, 2, 3) or a Matrigel matrix (lane 4). Western blotting panels shown in this figure are a representative of three independent experiments. (**B**) The levels of phosphorylated EGFR (Y1045), total EGFR, phosphorylated HER2 (Y1248) and total HER2 were examined in WCL of Parental JIMT1 and T-DM1R cells treated with either 4 µg/mL T-DM1, 100 ng/mL EGF, 5 µM erlotinib, or left untreated for 48 h. Western blotting panels shown in this figure are a representative of three independent experiments. (**C**) The levels of phosphorylated HER2 (Y1248) and total HER2 expression were examined in WCL of either 5 µM erlotinib-treated or untreated parental JIMT1 cells cultured on a Matrigel matrix for 48 h. Western blotting panels shown in this figure are a representative of three independent experiments. (**D**) The levels of phosphorylated HER2 (Y1248) and total HER2 expression were examined in WCL of control and HER2 siRNA-treated parental JIMT1 cells, and control and EGFR siRNA-treated T-DM1R cells by Western blot analysis. Western blotting panels shown in this figure are a representative of three independent experiments. (**E**) Upper panels: Fluorescent immunostaining images showing T-DM1 and HER2 in parental JIMT1 cells incubated with or without 100 ng/mL EGF overnight and on the next day treated with 100 µg/mL T-DM1 for 4 h. Scale bar: 50 µm. Lower panels: Fluorescent immunostaining images showing T-DM1 and LAMP-1 in parental JIMT1 cells incubated with or without EGF and treated with 100 µg/mL T-DM1 for 4 h. Scale bar: 20 µm.

We next explored the mechanisms that may cause parental JIMT1 cells to lose their sensitivity to T-DM1 and form spheroids on the Matrigel matrix. Because it is difficult to measure the total amount of protein harvested from spheroids, we seeded parental JIMT1 cells at different densities in 2D cell culture dishes to ensure the total amount of whole cell lysate (WCL) would be comparable to the total amount of protein harvested from spheroids. Figure 2A (lanes 1, 2 and 3) shows the amount of WCL harvested from three different plated cell densities of parental JIMT1 cells. We examined the expression levels of EGFR and HER2, and their phosphorylated forms in the WCL collected from cells cultured in 2D and spheroids. As shown in Figure 2A, the level of EGFR was dramatically decreased in spheroids compared to parental JIMT1 cells grown in 2D dishes (compare lane 2 with lane 4, both lanes loaded with similar amount of total protein). In contrast, the level of phosphorylated EGFR or the ratio of phosphorylated EGFR/total EGFR in spheroids was dramatically increased as compared to that in cells cultured in 2D cell culture dishes (5.6-fold increase, lane 4 compared to lane 2). It has been well established that EGF induces EGFR activation and transduces growth and survival signals to downstream pathways such as ERK1/2, accompanied by rapid EGFR degradation [21,22]. Data shown in Figure 2A indicates that when T-DM1 sensitive JIMT1 cells were seeded in a different tumor microenvironment, such as Matrigel, EGFR and the downstream signaling pathways of EGFR were activated. This EGFR activation followed by the receptor downregulation in spheroids mimics EGF-mediated receptor activation and downregulation. We previously reported that upregulation of EGFR was observed in the acquired T-DM1-resistant JIMT1 cells, T-DM1R, and inhibition of EGFR activity by erlotinib, a tyrosine kinase inhibitor of EGFR, inhibited T-DM1R cell growth and rendered T-DM1R cells to be sensitive to T-DM1 treatment [12]. We also observed a 3.7-fold increase in phosphorylated HER2 in spheroids compared to cells cultured in 2D dishes although total HER2 remained unchanged in spheroids (lane 2 versus lane 4 in Figure 2A). In addition, ERK activity was enhanced in spheroids (2.2-fold increase, lane 4 compared to lane 2), which provides additional evidence indicating that the EGFR signaling pathway is activated in spheroids (Figure 2A). Taken together, data shown in Figure 2A indicates that the Matrigel matrix can activate EGFR signaling in JIMT1 cells, which may lead to loss of T-DM1 sensitivity.

Figure 2B showed the changes in the levels of EGFR and HER2 in parental JIMT1 and T-DM1R cells treated with T-DM1, EGF, erlotinib, or left untreated for 48 hrs. The levels of total EGFR in T-DM1R cells were substantially higher than that in parental JIMT1 cells (top panel, Figure 2B). While phosphorylated EGFR was not significantly increased in either parental JIMT1 or T-DM1R in the cells treated with EGF (Note: The cells were incubated in 10 % FBS containing media), the levels of total EGFR was reduced in both parental JIMT1 and T-DM1R cells treated with EGF as compared to that in the none-treated cells (Figure 2B). This resulted in the dramatical increase in ratio of phosphorylated EGFR/total EGFR in JIMT1 and T-DM1R cells treated with EGF, indicating that the ligand (EGF) induces receptor activation and degradation. Furthermore, the ratio of phosphorylated HER2/total HER2 was also increased in both parental JIMT1 and T-DM1R cells treated with EGF as compared to non-treated cells, suggesting that HER2 was also activated when the cells were treated with EGF (Figure 2B). Erlotinib reduced the amount of phosphorylated EGFR and HER2 in both cell types, indicating that EGFR kinase activity is necessary for maintaining HER2 protein expression and activity in both parental and T-DM1R cells (Figure 2B). Similar to parental JIMT1 cells in the 2D system, erlotinib was also capable of inhibiting both EGFR and HER2 phosphorylation in spheroids in the Matrigel system (Figure 2C and Figure S1A).

The functional correlation between EGFR and phosphorylated HER2 was further examined using siRNA technology. Parental JIMT1 cells express high levels of HER2 with low levels of EGFR, whereas T-DM1R cells express low levels of HER2 and high levels of EGFR (Figure 2B,D). Supplemental Figure S1C,D showed the efficiency of HER2 and EGFR knock-down in parental JIMT1 and T-DM1R cells, respectively. Silencing HER2 did not change the level of phosphorylated EGFR in the parental cells (Figure S1D). In contrast, the level of phosphorylated HER2 was dramatically decreased in EGFR knock-downed T-DM1R cells (Figure 2D). These results further confirm that EGFR activity is necessary to maintain HER2 activity in cells and support the idea that EGFR is activated in spheroids, resulting in increased HER2 phosphorylation.

To address the insensitivity of parental JIMT1 cells to T-DM1 treatment in the Matrigel, we examined the accessibility of T-DM1 to parental JIMT1 cells seeded in 2D dishes. Parental JIMT1 cells were seeded on glass coverslips treated with EGF or left untreated. After 24 h, cells were incubated with 100 µg/mL T-DM1 for 4 h, and then subjected to fluorescent immunostaining for T-DM1, HER2 and a lysosomal marker LAMP-1. Figure 2E showed that cell-bound T-DM1 and co-localization of T-DM1 and HER2 were immunodetected in non-EGF treated parental JIMT1 cells in the 2D system. In contrast, in EGF-treated cells, the amount of T-DM1 detected was reduced (Figure 2E, comparing EGF-treated and non-EGF-treated images in the first column), which was expected as HER2 expression in EGF-treated cells was decreased (Figure 2B). Furthermore, the co-localization of T-DM1 and HER2 was also reduced in the EGF-treated cells compared to non-EGF-treated cells (Figure 2E, upper two panels). In addition, co-localization of T-DM1 and LAMP-1 was immunodetected in non-EGF treated cells (Figure 2E, arrows in the third panel from the top). These results indicate that downregulation of HER2 induced by the activation of EGFR decreased the binding of T-DM1 to HER2 on the cell surface, leading to the reduced T-DM1-mediated internalization of T-DM1/HER2 complex into the lysosomal pathway.

### 2.3. Combination of T-DM1 with Erlotinib Kills JIMT1-Spheroids on Matrigel

**Figure 3:**
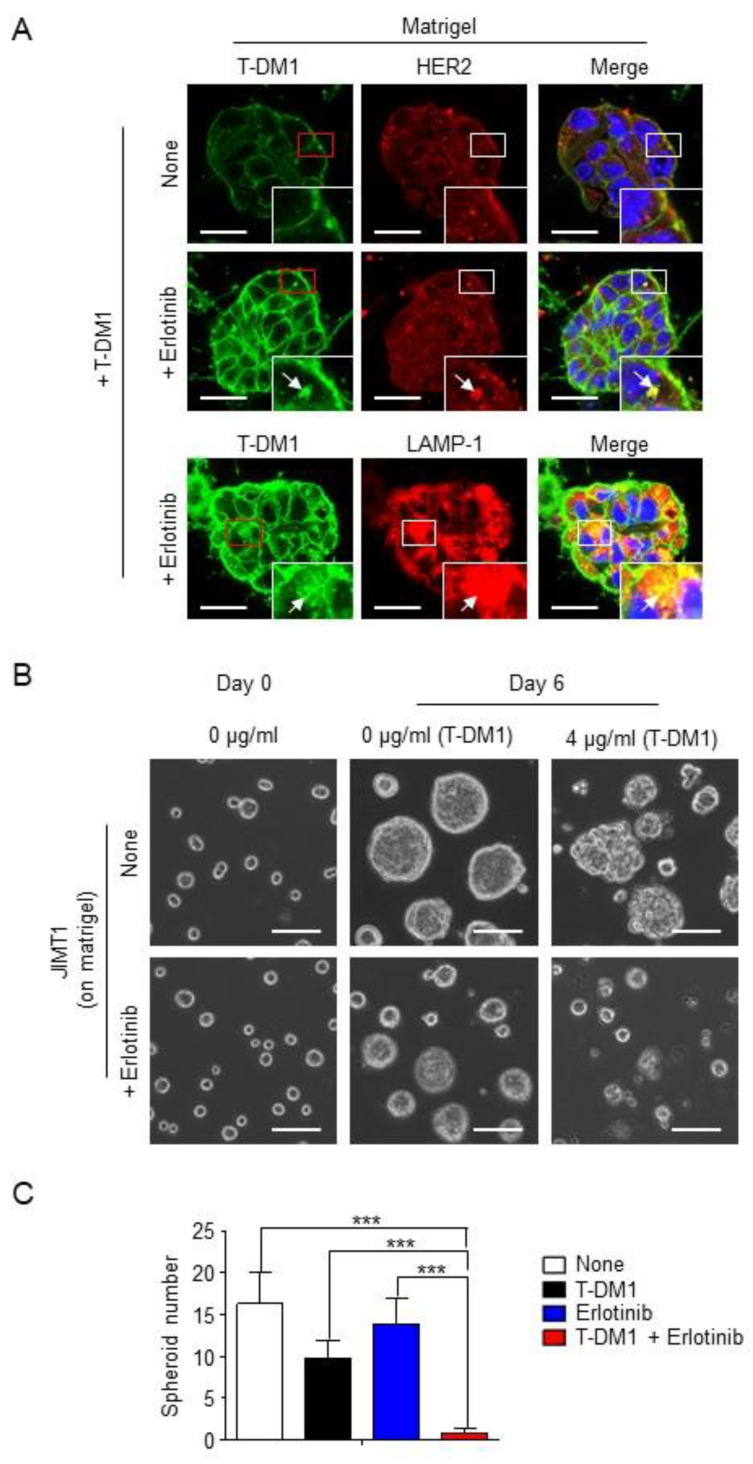
Blocking EGFR tyrosine kinase activation by erlotinib allows T-DM1 binding to HER2 and kills JIMT1-spheroids on the Matrigel matrix. (**A**) Upper panels: Fluorescent immunostaining images showing T-DM1 and HER2 in parental JIMT1 cell-derived spheroids cultured on a Matrigel, incubated with or without 5 µM erlotinib overnight and treated with 100 µg/mL T-DM1 for 2 h. Scale bar: 20 µm. Lower panels: Fluorescent immunostaining images showing T-DM1 and LAMP-1 in either 5 µM erlotinib or left untreated parental JIMT1 cell-derived spheroids cultured on a Matrigel, incubated with or without 5 µM erlotinib overnight and treated with 100 µg/mL T-DM1 for 2 h. Scale bar: 20 µm. (**B**) Bright field images showing parental JIMT1 cells treated with 4 µg/mL T-DM1, 5 µM erlotinib, T-DM1 + erlotinib, or left untreated cultured on a Matrigel matrix (Day 6). Scale bar: 100 µm. (**C**) Growth profiles of parental JIMT1-spheroids (radius >20 µm) parental JIMT1treated with 4 µg/mL T-DM1, 5 µM erlotinib, T-DM1 + erlotinib, or left untreated cultured on a Matrigel matrix. Spheroid number per image (area of 0.325 mm^2^) was counted. ***: *p* < 0.0001.

**Figure 4:**
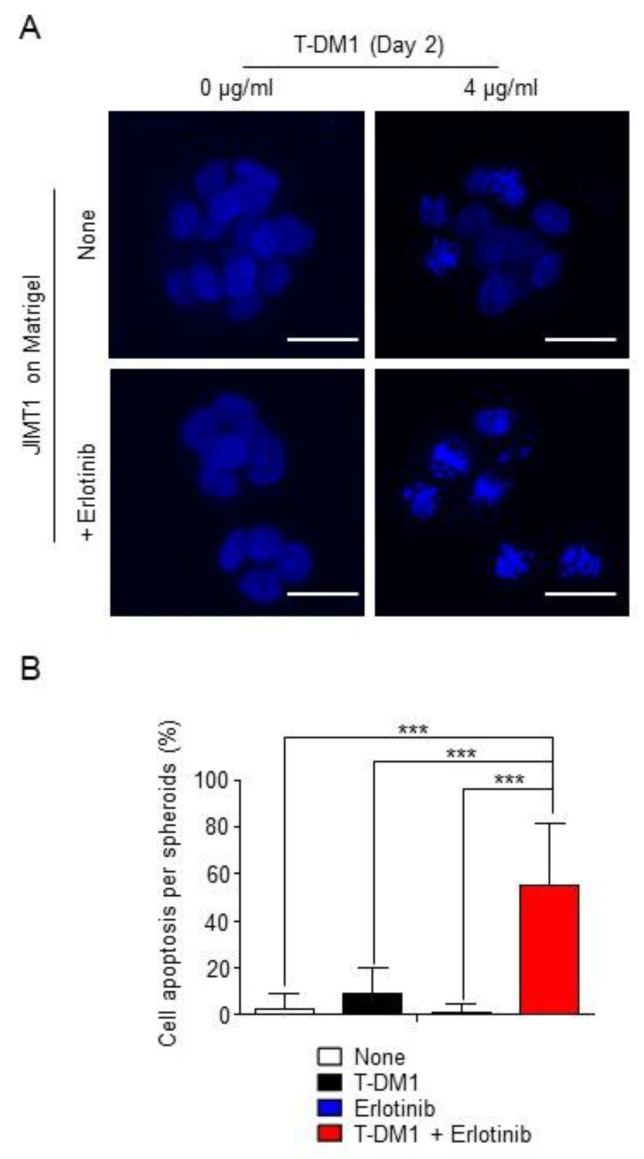
Blocking EGFR tyrosine kinase activation by erlotinib allows T-DM1 to induce apoptosis of JIMT1 cell spheroids on the Matrigel matrix. (**A**) Nuclear staining images showing parental JIMT1 cells cultured on a Matrigel matrix and treated with 4 µg/mL T-DM1, 5 µM erlotinib, T-DM1 + erlotinib, or left untreated. Images were taken on Day 3. Scale bar: 20 µm. (**B**) Cell apoptosis profiles in parental JIMT1 cell spheroids treated with 4 µg/mL T-DM1, 5 µM erlotinib, T-DM1 + erlotinib, or left untreated cultured on a Matrigel matrix. Data were collected on Day 3. Total 20 spheroids in each treatment were randomly picked up and examined. ***: *p* < 0.0001.

We next asked the question of whether inhibition of EGFR activity in spheroids enhances T-DM1 binding to the cell surface via HER2. Figure 3A shows that cell morphology in the spheroid on the Matrigel appears to be round and tightly associated. While a weak florescent signal from T-DM1 was detected in the spheroids, co-localization between T-DM1 and HER2 was not detected in the spheroids in the non-erlotinib-treated cells (Figure 3A, upper panel). We also examined the co-localization between T-DM1 and HER2 using the ECM gel, which can also support the cells form spheroids on the gel, as a control to the Matrigel. The co-localization between T-DM1 and HER2 was also not observed in the spheroids on the ECM gel, whereas the co-localization was strongly observed in cells on fibronectin (Figure S2A,B). In contrast, when the spheroids were treated with erlotinib, the amount of cell-surface bound T-DM1 was dramatically increased, and co-localizations of T-DM1 with HER2 and LAMP-1 were strongly enhanced (Figure 3A, bottom panel). Furthermore, as a result of the increased amount of cell-surface bound T-DM1, T-DM1 was able to inhibit parental JIMT1 cells from forming spheroids on the Matrigel when combined with erlotinib, whereas neither T-DM1 nor erlotinib was capable of inhibiting these cells from growing and forming spheroids (Figure 3B,C). These results provide strong evidence that EGFR activation followed by HER2 phosphorylation plays an important role in controlling cell sensitivity to T-DM1. These results were further confirmed by a nuclear staining of spheroids with DAPI. Figure 4A,B show that combination of T-DM1 with erlotinib was able to induce nuclear fragmentation or apoptosis in more than 50% of cells in the spheroids grown on the Matrigel, whereas T-DM1 alone only moderately caused the nuclear fragmentation, and erlotinib was not capable of causing nuclear fragmentation. Taken together, these data demonstrate that extrinsic factors from the tumor microenvironment can alter tumor cell sensitivity to T-DM1, resulting in the primary resistance to T-DM1.

### 2.4. SKBR-3 Cells Are Less Sensitive to T-DM1 and Capable of Developing Spheroids on the Matrigel 

**Figure 5:**
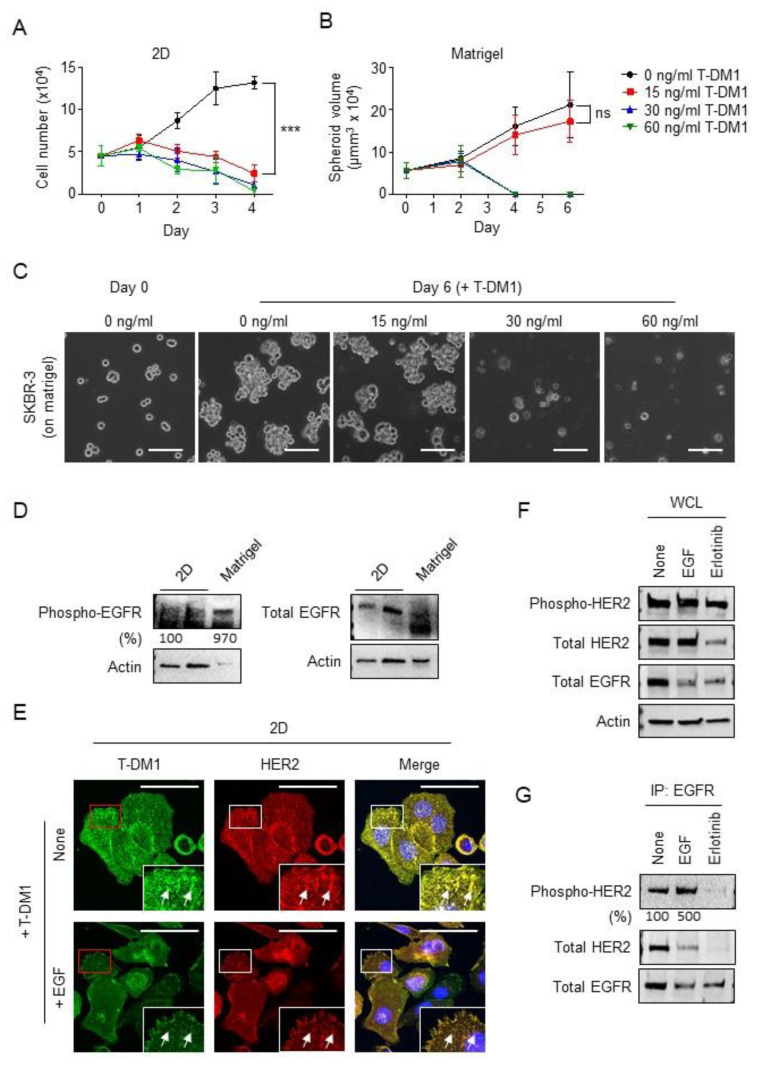
EGFR activation followed by HER2 phosphorylation blocks T-DM1 accessibility to cell surface HER2 in SKBR-3 cells cultured on the Matrigel matrix and on 2D in the presence of EGF. (**A**) Cell growth profiles of SKBR-3 cells treated with four different concentrations of T-DM1 (0, 15, 30, and 60 ng/mL) on 2D cell culture system. ***: *p* = 0.0001 (**B**) Grow profiles of SKBR-3 cell spheroids (radius ≥20 µm) treated with four different concentrations of T-DM1 (0, 15, 30, and 60 ng/mL) on a Matrigel matrix. No significance (ns): *p* = 0.2235 (**C**) Bright field images of SKBR-3 cells cultured in the absence or presence of 15, 30, and 60 ng/mL T-DM1 on a Matrigel matrix. Scale bar: 100 µm. (**D**) The levels of phosphorylated EGFR (Y1045) and total EGFR were examined in WCL of SKBR-3 cells cultured on a Matrigel matrix or 2D. Western blotting panels shown in this figure are a representative of two independent experiments. (**E**) Fluorescent immunostaining images showing T-DM1 and HER2 in SKBR-3 cells incubated with or without 100 ng/mL EGF overnight and treated with 100 µg/mL T-DM1 for 2 h on 2D cell culture. Scale bar: 50 µm. (**F**) The levels of phosphorylated HER2 (Y1248), total HER2, and total EGFR were examined in WCL of SKBR-3 cells treated with either 100 ng/mL EGF, 5 µM erlotinib, or left untreated for 24 h. Western blotting panels shown in this figure are a representative of three independent experiments. (**G**) WCLs of SKBR-3 cells treated as indicated were subjected to co-immunoprecipitation experiments using an anti-EGFR antibody, and the phosphorylated HER2 (Y1248) in anti-EGFR immunoprecipitates was analyzed by Western blot. Western blotting panels shown in this figure are a representative of three independent experiments.

We next examined the functional correlation between EGFR activation and the sensitivity to T-DM1 in a different breast cancer cell line, SKBR-3, a HER2-positive breast cancer cell line that is very sensitive to T-DM1 [23]. As shown in Figure 5A, SKBR-3 cells were about 130 times more sensitive to T-DM1 than JIMT1 cells (IC50 for SKBR-3: 15 ng/mL versus IC50 for JIMT1: 2 µg/mL, data not shown). Almost all the cells died when incubated in cell culture media containing 15 ng/mL of T-DM1 for 4 days on the 2D culture system (Figure 5A). In contrast, in the Matrigel system SKBR-3 cells survived 6 days in the presence of 15 ng/mL of T-DM1 and grew and formed spheroid-like clusters (Figure 5B,C). These results demonstrate that

SKBR-3 cells were less sensitive to T-DM1 or more resistant to a relatively low dose of T-DM1 on the Matrigel compared to that in 2D cell culture dishes. Similar to data shown in Figure 2A, we also found that the phosphorylation of EGFR was increased about 9.7-fold and total EGFR was downregulated in SKBR-3 cells cultured on Matrigel compared to the SKBR-3 cells grown in 2D cell culture dishes (Figure 5D), indicating that EGFR activation also plays a critical role in rendering SKBR-3 cells resistant to T-DM1 at 15 ng/mL on Matrigel. We further explored whether EGFR activation might contribute to T-DM1 resistance of SKBR-3 cells and examined co-localization of T-DM1 with HER2 on the cell surface in the 2D system. As shown in Figure 5E, a significant amount of T-DM1 was detected in non-EGF-treated SKBR-3 cells and co-localization between T-DM1 and HER2 was immunodetected. In contrast, the amount of T-DM1 on cell surface and the co-localization between T-DM1 and HER2 were dramatically reduced in the EGF-treated cells (Figure 5E, arrows).

To investigate the mechanism by which EGF reduces binding of T-DM1 to cell surface HER2, which may lead to HER2-positive breast cancer cells resistance to T-DM1, we examined protein levels of HER2 in SKBR-3 cells treated with EGF, erlotinib or left untreated. Figure 5F shows that neither HER2 expression nor phosphorylation were reduced in EGF-treated SKBR-3 cells compared to non-treated SKBR-3 cells. As expected, the level of EGFR protein expression was reduced in EGF-treated SKBR-3 cells, consistent with the results from parental JIMT1 and T-DM1R cells (Figure 2B). Figure 5F showed that the total EGFR was reduced in erlotinib-treated SKBR-3 cells. We next examined the interaction between phosphorylated HER2 and EGFR using a co-immunoprecipitation experiment. As shown in Figure 5G, the amount of phosphorylated HER2 immunoprecipitated by EGFR in EGF-treated cells was more than that in non-treated cells (top panel). These data suggest that EGFR likely forms a heterodimer with phosphorylated HER2 induced by EGF. This heterodimer formation may contribute to HER2 reduction on the cell surface since EGF mediates receptor internalization followed by the lysosomal degradation. This may lead to the reduced amount of HER2 on the cell surface available to associate with T-DM1. In contrast, erlotinib inhibits the kinase activity of EGFR, and abolished the interaction between EGFR and HER2, which enables T-DM1 to bind to cell surface HER2, therefore, enhances T-DM1-mediated cytotoxicity. Taken together, these results confirm that phosphorylation of HER2 induced by the activation of EGFR reduced binding of T-DM1 to HER2 on the cell surface in SKBR-3 cells.

## 3. Discussion

The tumor microenvironment promotes not only tumor progression but also drug resistance [4]. However, it remains largely unclear how the tumor microenvironment specifically promotes drug resistance. It has been reported that tumor cell-extrinsic factors derived from the tumor microenvironment promote an epithelial-to-mesenchymal transition (EMT) in a subpopulation of tumor cells, and subsequently the EMT enables cell plasticity to increase the chance to become target drug resistant without genetic changes [1,4,24]. EMT is demonstrated by typical cell morphological changes such as losing cell-to-cell junctions, remodeling the cytoskeleton, interacting with ECM, and cell polarity changes [4]. In this study, we showed that changes in the tumor microenvironment, using Matrigel as cell culture media, caused T-DM1 resistance of HER2-positive breast cancer cells that are otherwise sensitive to T-DM1 treatment in 2D cell culture dishes. This resistance of breast cancer cells to T-DM1 was not induced by the conventional approach to chronically expose T-DM1-sensitive cells to the escalating doses of T-DM1.

This study provides several insights into mechanisms that may contribute to the primary resistance of parental JIMT1 and SKBR-3 cells to T-DM1. First, both parental JIMT1 and SKBR-3 cells were unable to spread when plated on the Matrigel and displayed a rounded cell morphology. This morphological change triggered by the tumor microenvironment may limit the access of T-DM1 to associate with the cell surface HER2. However, we found that erlotinib-treated spheroids became sensitive to T-DM1 and the structure of these spheroids appeared to be maintained, suggesting that the cell morphological changes through an EMT-like process may not be a major factor in the loss of parental JIMT1 cell sensitivity to T-DM1 on the Matrigel matrix. Secondly, the tumor cell-extrinsic factors from the Matrigel activate ErbB family receptors, EGFR and HER2. This leads to the upregulation of signaling pathways downstream of EGFR and HER2, such as ERK activity, which in turn produces strong mitogenic signaling to allow parental JIMT1 cells to grow and form spheroids in the presence of T-DM1. Thirdly, activation and tyrosine phosphorylation of EGFR and HER2 result in receptor internalization followed by degradation in the cells of spheroids. This reduces the level of HER2 presented on the cell surface and further reduces T-DM1 access to associate with HER2. This proposed mechanism is underscored by the finding that erlotinib treatment inhibits tyrosine phosphorylation of both EGFR and HER2, elevates the levels of cell surface HER2, renders spheroids sensitive to T-DM1, and exhibits inhibitory effect on growth of spheroids with T-DM1. Fourthly, similar data obtained from SKBR-3 cells provide additional evidence to support the idea that changes in tumor microenvironments can activate intracellular growth signaling pathways and reduce sensitivity of HER2-positive breast cancer cells that are otherwise very sensitive to T-DM1 in 2D cell culture dishes. Lastly, it has been reported that trastuzumab, the antibody portion of T-DM1, preferentially blocks HER2 homodimer formation, but not heterodimer [25]. Our data also show that EGFR activation can drive the formation of EGFR/HER2 heterodimers, leading to the tyrosine phosphorylation of HER2. Based on this information, we speculate that T-DM1 may not effectively associate with phosphorylated HER2 in EGFR/HER2 heterodimers induced by EGF, preventing T-DM1/HER2 complex formation and internalization followed by lysosomal degradation. This results in reduced sensitivity of parental JIMT1 and SKBR-3 cells to T-DM1.

Collectively, we provide a novel insight into how cancer cells interact with the tumor microenvironment and how the extrinsic factors lead to primary resistance to T-DM1. Our discovery of tumor microenvironment controlling the sensitivity to T-DM1 through upregulating EGFR activity provides an opportunity to develop a new therapeutic strategy to overcome primary resistance to T-DM1.

## 4. Materials and Methods 

### 4.1. Cells, Therapeutic Drugs and Matrigel/ECM Gel

A primary trastuzumab-resistant breast cancer cell line JIMT1 and JIMT1-derived T-DM1-resistant cells (designated as T-DM1R) were maintained as described previously [12]. SKBR-3 cells were purchased from ATCC, and they were maintained in DMEM/F12 (1:1) media containing 10% FBS. T-DM1 and erlotinib were purchased from the pharmacy (Bethesda, NIH, Bethesda, MD, USA) and Selleckchem (Houston, TX, USA, cat# S1023), respectively. Matrigel matrix (Corning, NY, USA, cat# 354234) and ECM gel (Sigma-Aldrich, St. Louis, MO, USA, cat# E1207), both of which can support the cells to form spheroids on the gel, were prepared according to the manufacturers’ instructions. Briefly, vials of aliquoted Matrigel or ECM gel stored in at −20 °C were thawed on ice and the liquid Matrigel or ECM gel was immediately applied to 12-well (about 400 µL per well) or 6-well plates (about 600 µL per well). The dishes were incubated at 37 °C for 30 min until the Matrigel or ECM gel was polymerized.

### 4.2. Cell Growth and Matrigel Assays

5 × 10^4^ parental JIMT1, T-DM1R or SKBR-3 cells were seeded in 12-well plates (Corning) and incubated in cell culture media containing 10% FBS overnight. In the next day, the cell culture media was changed to fresh media containing T-DM1 or none as a control. The cell culture media was changed to the fresh media containing T-DM1 or none as a control every other day. For counting cells on 2D, the cells were washed with PBS, and then were trypsinized with 100 µL of 0.05% trypsin/EDTA (Thermo Fisher Scientific, Waltham, MA, USA). Subsequently, 100 µL of 0.4% trypan blue/0.85% NaCl (Lonza, Basel, Switzerland) was added to the well, and cell number was counted using a TC20 automated cell counter (Bio-Rad, Hercules, CA, USA). 1 × 10^5^ or 5 × 10^5^ JIMT1 or SKBR-3 cells were seeded on the top of the polymerized Matrigel in 12-well or 6-well plates and incubated in the cell culture media containing T-DM1 or none as the control, and the cell culture media were then changed every other day. Cells and spheroids cultured on 12-well and 6-well plates were subjected to bright field imaging and Western blot analysis. 4–6 pictures/well were randomly taken on day 0, 4 and 6. The number of spheroids per image (area of 0.325 mm^2^) were counted, and the volumes of the spheroids were measured using Image J and calculated using the following formula:

4/3 × π × (radius)^3^

### 4.3. Immunofluorescent Staining

The detailed experimental procedures were described previously [12]. Briefly, cells were plated on glass coverslips in 12-well plates or seeded on either Matrigel, ECM gel or fibronectin (Sigma-Aldrich, cat# F1141) in MatTek dishes (MatTek corporation, Ashland, MA, USA, cat# P35GC-1.5-10-C). Parental JIMT1 or SKBR-3 cells were then fixed in 100% methanol at −20 °C for 20 min. After blocking with 10% donkey serum (Jackson ImmunoResearch, West Grove, PA, USA, cat# 017-000-121) at room temperature for 1–2 h, cells were subjected to fluorescent immunostaining. ProLong Gold antifade reagent with DAPI (Thermo Fisher Scientific, cat# P36935) was used for mounting specimens on glass slides and nuclear staining. Images were captured by a LSM880 confocal microscope (Carl Zeiss Microscopy, White Plains, New York, USA). The following primary antibodies were used: HER2 (Cell Signaling Technology, Danvers, MA, USA, cat# 2165) and LAMP-1 (BD Pharmingen, San Jose, CA, USA, cat# 555798). The following secondary antibodies were used: DyLight 488-conjugated anti-human IgG (Thermo Fisher Scientific, cat# SA5-10126), Alexa Fluor 549-conjugated anti-rabbit IgG (Thermo Fisher Scientific, cat# A21121) and Alexa Fluor 549-conjugated anti-mouse IgG (Thermo Fisher Scientific, cat# A21203).

### 4.4. Western Blotting and Immunoprecipitation

5 × 10^5^ parental JIMT1, T-DM1R JIMT1 cells, or SKBR-3 cells were seeded in 6-well plates one day prior to cell treatment. In the next day, the cells were treated with indicated drugs as described in the manuscript. 24 h or 48 h post drug treatment, the cells were washed with PBS twice and then lysed with a NP40 lysis buffer on ice for 30 min. After centrifugation, the whole cell lysates (WCL) were subjected to Western blot analysis. For immunoprecipitation of EGFR shown in Figure 5B, WCLs were incubated with EGFR antibody (Cetuximab, the NIH Pharmacy, Bethesda, MD, USA) at 4 °C for 2 h. The WLCs were subjected to immunoprecipitation using Protein A agarose (Sigma-Aldrich, cat# P140G) to capture cetuximab after incubation at 4 °C for 2 h. After washing the Protein A agarose beads with the lysis buffer, the immunoprecipitates were eluted. The immunoprecipitated EGFR was then detected using Western blotting using anti-EGFR antibody. The following primary antibodies were used for Western blotting analysis: EGFR (BD Biosciences, cat# 610016), phospho-EGFR (Y1045) (Cell Signaling Technology, cat# 2237), ERK1/2 (Cell Signaling Technology, cat# 4695), phosphor-ERK1/2 (Cell Signaling Technology, cat# 9106), HER2 (Cell Signaling Technology, cat# 2165), phospho-HER2 (Y1248) (Sigma-Aldrich, cat# SAB43000061), and Actin (Sigma-Aldrich, cat# A2066). Image J software (NIH, Bethesda) was used for densitometry of Western blotting. 

### 4.5. siRNA Transfection

The detailed procedures were previously described [12]. Briefly, one day prior to siRNA transfection, 1 × 10^5^ parental JIMT1 or T-DM1R cells were seeded in 6-well plates. In the next day 25 nM siRNA was transfected using Lipofectamine 3000 (Thermo Fisher Scientific, cat# L3000-015) and 96 h post transfection, the cells were subjected to Western blot analysis. The following siRNAs, purchased from Dharmacon (Lafayette, CO, USA), were used in the manuscript: human HER2 (cat# L-003126-00-0005, siRNAs: UGGAAGAGAUCACAGGUUA, GAGACCCGCUGAACAAUAC, GGAGGAAUGCCGAGUACUG, GCUCAUCGCUCACAACCAA), human EGFR (cat# L-003114-00-0005, siRNAs: CAAAGUGUGUAACGGAAUA, CCAUAAAUGCUACGAAUAU, GUAACAAGCUCACGCAGUU, CAGAGGAUGUUCAAUAACU) and non-targeting control (cat# D-001819-10-20, siRNAs: UGGUUUACAUGUCGACUAA, UGGUUUACAUGUUGUGUGA, UGGUUUACAUGUUUUCUGA, UGGUUUACAUGUUUUCCUA).

### 4.6. Statistical Analysis

GraphPad Prism (GraphPad Software, Inc., Jolla, CA, USA) was used for statistical studies. Statistical significance was determined by Student’s *t*-test (*, *p*-value < 0.05; **, *p*-value < 0.01; ***, *p*-value < 0.0001). Data is expressed as mean ± SD.

## 5. Conclusions

In summary, we demonstrated that HER2-positive, T-DM1-sensitive breast cancer cells exhibited resistant or less sensitive to T-DM1 and were able to form spheroids in the present of T-DM1 when cultured on the Matrigel. On Matrigel, EGFR was activated in these cells-derived spheroids, and the EGFR activation leaded to HER2 phosphorylation and degradation, resulting in the resistance or the reduced sensitivity to T-DM1. These results demonstrate that extrinsic factors from the tumor microenvironment control the sensitivity of HER2-positive breast cancer cells to T-DM. The tyrosine kinase inhibitor of EGFR, however, can reverse the sensitivity of these cells to T-DM1. Our discovery suggests that the combination of EGFR kinase inhibitor with T-DM1 may be a promising novel therapeutic strategy to overcome primary T-DM1 resistance and warrants further pre-clinical and clinical investigation for the treatment of HER2-positive breast cancers. 

## Data Availability

The data presented in this study are available in this article and supplementary materials.

## Supplementary Materials

The following are available online at https://www.mdpi.com/article/10.3390/cancers13102331/s1. Figure S1: (A) The levels of phosphorylated EGFR and total EGFR were evaluated by Western blot of WCL of 5 µM erlotinib-treated and untreated parental JIMT1 cells cultured on a Matrigel matrix for 48 h, (B) The levels of total HER2 expression were examined in WCL of control and HER2 siRNA-treated parental JIMT1 cells, and control and EGFR siRNA-treated T-DM1R cells by Western blot analysis. (C) The levels of total EGFR expression were examined in WCL of control and HER2 siRNA-treated parental JIMT1 cells, and control and EGFR siRNA-treated T-DM1R cells by Western blot analysis. (D) The levels of phosphorylated EGFR (Y1045) and total EGFR expressions were examined in WCL of control and HER2 siRNA-treated parental JIMT1 cells, and control and EGFR siRNA-treated JIMT1-T-DM1R cells by Western blot analysis. Figure S2: Co-localization of T-DM1 and HER2 in cells cultured on the ECM proteins (ECM Gel and fibronectin) as additional controls to Matrigel. (A) Fluorescent immunostaining images showing T-DM1 and HER2 in parental JIMT1 cell-derived spheroids cultured on the ECM Gel for 3 days and treated with 100 µg/mL T-DM1 for 5 h. Scale bar: 20 µm. (B) Upper panels: Fluorescent immunostaining images showing T-DM1 and HER2 in parental JIMT1 cells cultured on fibronectin and treated with 100 µg/mL T-DM1 for 5 h. Scale bar: 50 µm. Lower panels: Fluorescent immunostaining images showing T-DM1 and LAMP-1 in parental JIMT1 cells cultured on fibronectin and treated with 100 µg/mL T-DM1 for 5 h. Scale bar: 50 µm. Figure S3: Unprocessed Western blot images for Figure 2A. Figure S4: Unprocessed Western blot images for Figure 2B.Figure S5: Unprocessed Western blot images for Figure 2C. Figure S6: Unprocessed Western blot images for Figure 2D. Figure S7: Unprocessed Western blot images for Figure 5D. Figure S8: Unprocessed Western blot images for Figure 5F and 5G. Figure S9: Unprocessed Western blot images for Figure S1A. Figure S10: Unprocessed Western blot images for Figure S1B–D.
