# Supplementary material for: Tumor Extrinsic Factors Mediate Primary T-DM1 Resistance in HER2-Positive Breast Cancer Cells
Source: Cancers (Basel). 2021 May 12;13(10):2331. doi: 10.3390/cancers13102331 (PMC8150545; doi:10.3390/cancers13102331)
Supplement: Supplementary file 1 [file cancers-13-02331-s001.zip › cancers-1182717-supplementary.pdf]

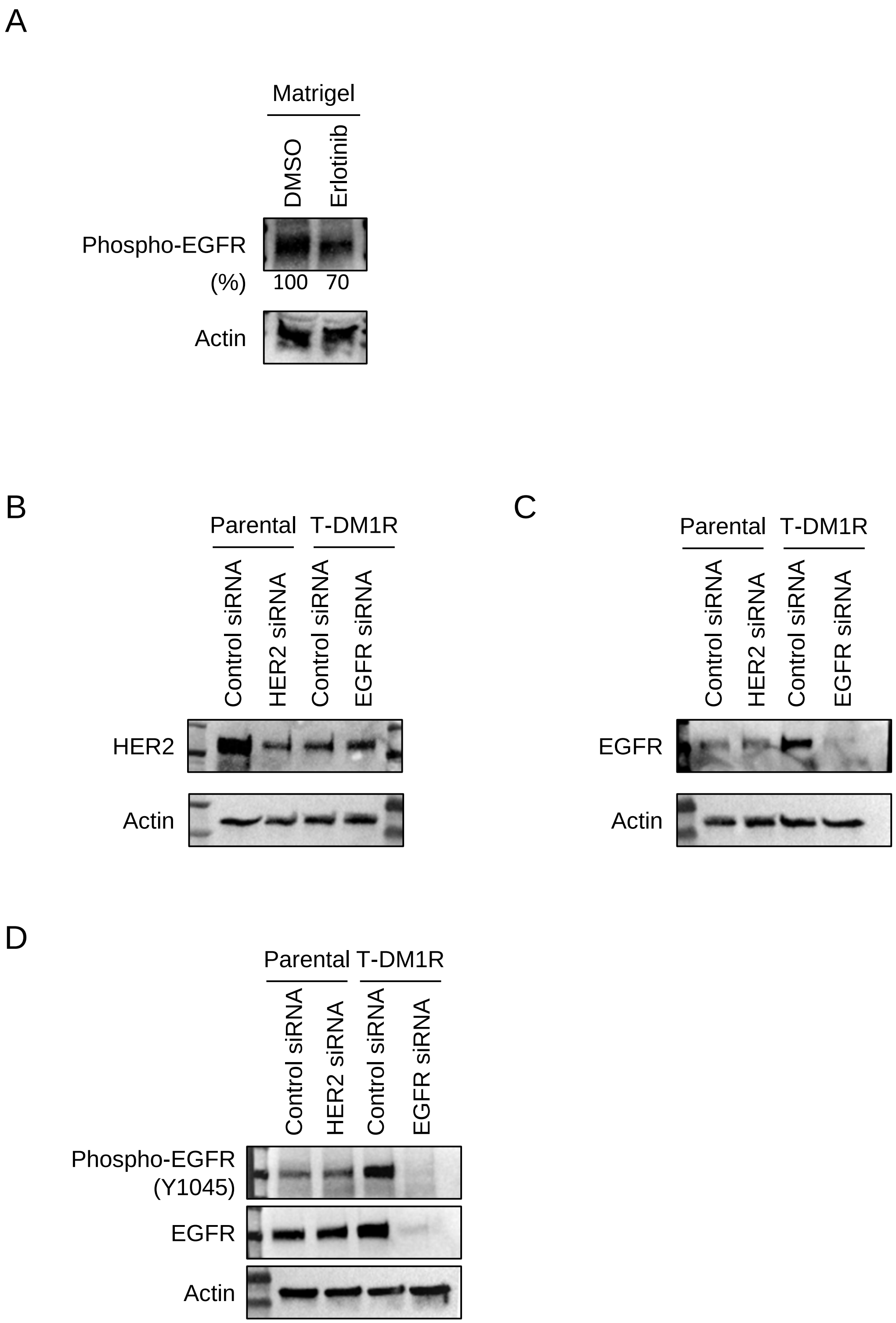
A
Matrigel
DMSO
Erlotinib
Phospho-EGFR
(%)
100
70
Actin
B
Parental
T-DM1R
Control siRNA
HER2 siRNA
Control siRNA
EGFR siRNA
HER2
Actin
C
Parental
T-DM1R
Control siRNA
HER2 siRNA
Control siRNA
EGFR siRNA
EGFR
Actin
D
Parental
T-DM1R
Control siRNA
HER2 siRNA
Control siRNA
EGFR siRNA
Phospho-EGFR
(Y1045)
EGFR
Actin

Figure S1

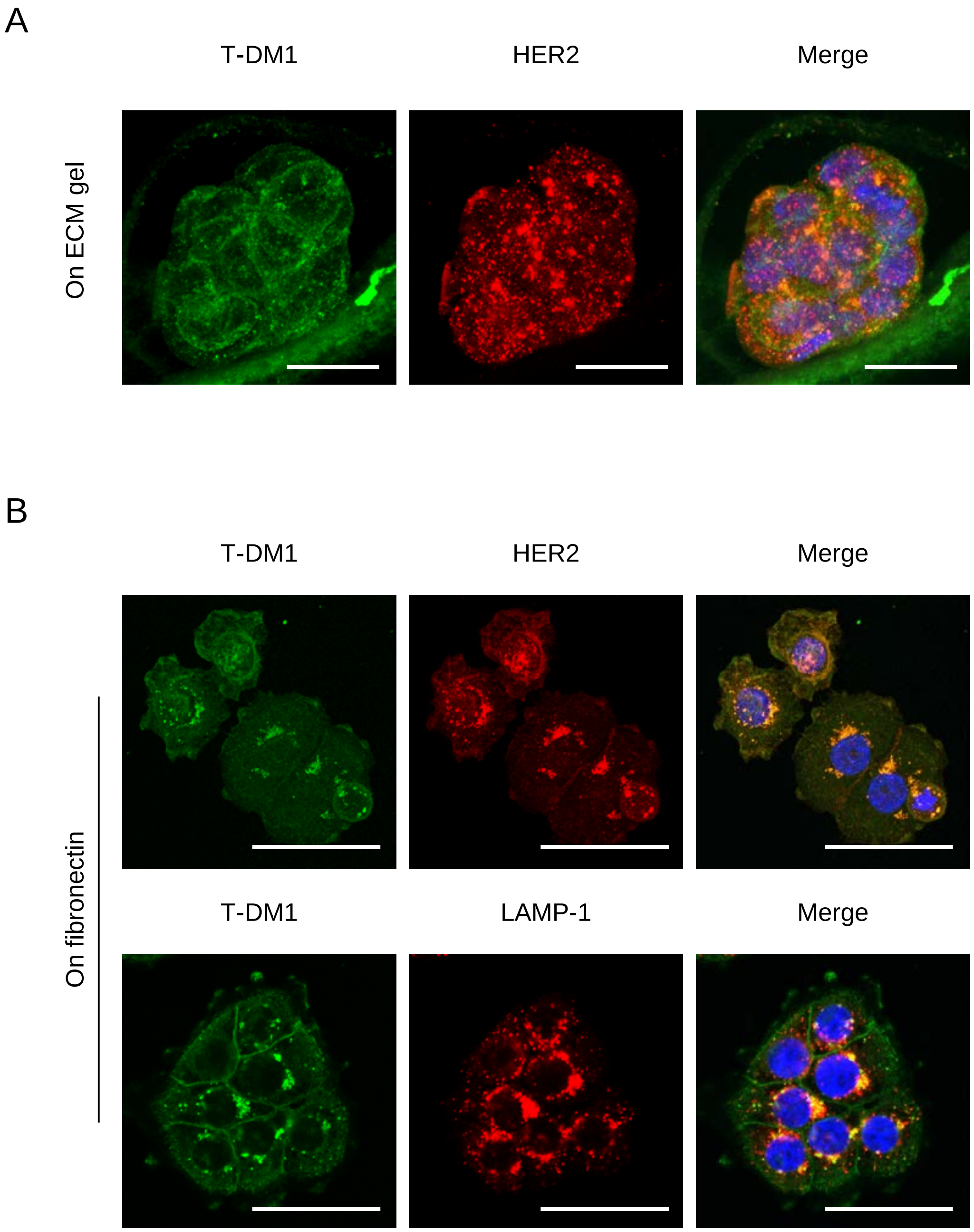
A
T-DM1
HER2
Merge
On ECM gel
B
T-DM1
HER2
Merge
On fibronectin
T-DM1
LAMP-1
Merge

Figure S2

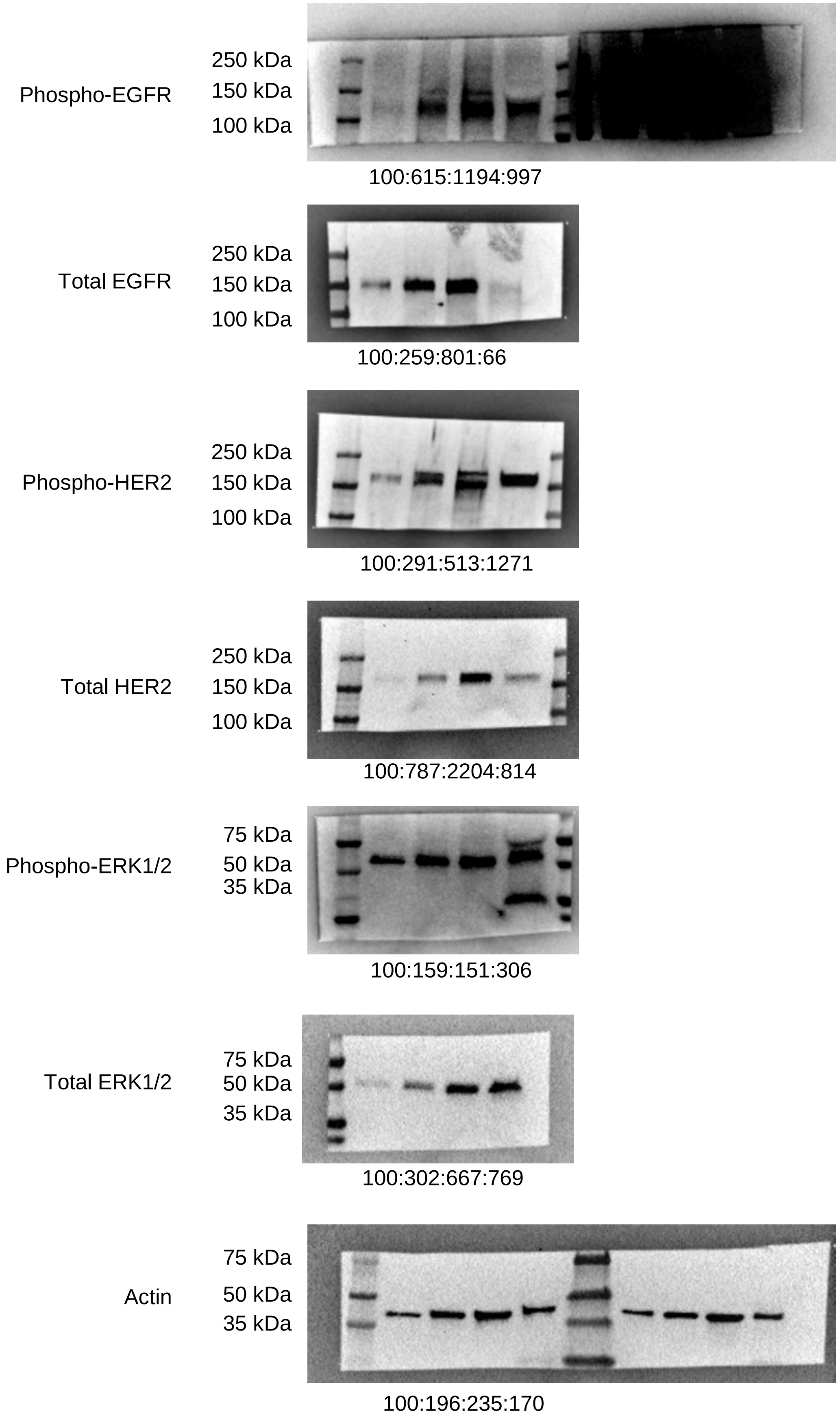
Phospho-EGFR
250 kDa
150 kDa
100 kDa
100:615:1194:997
Total EGFR
250 kDa
150 kDa
100 kDa
100:259:801:66
Phospho-HER2
250 kDa
150 kDa
100 kDa
100:291:513:1271
Total HER2
250 kDa
150 kDa
100 kDa
100:787:2204:814
Phospho-ERK1/2
75 kDa
50 kDa
35 kDa
100:159:151:306
Total ERK1/2
75 kDa
50 kDa
35 kDa
100:302:667:769
Actin
75 kDa
50 kDa
35 kDa
100:196:235:170

Figure S3

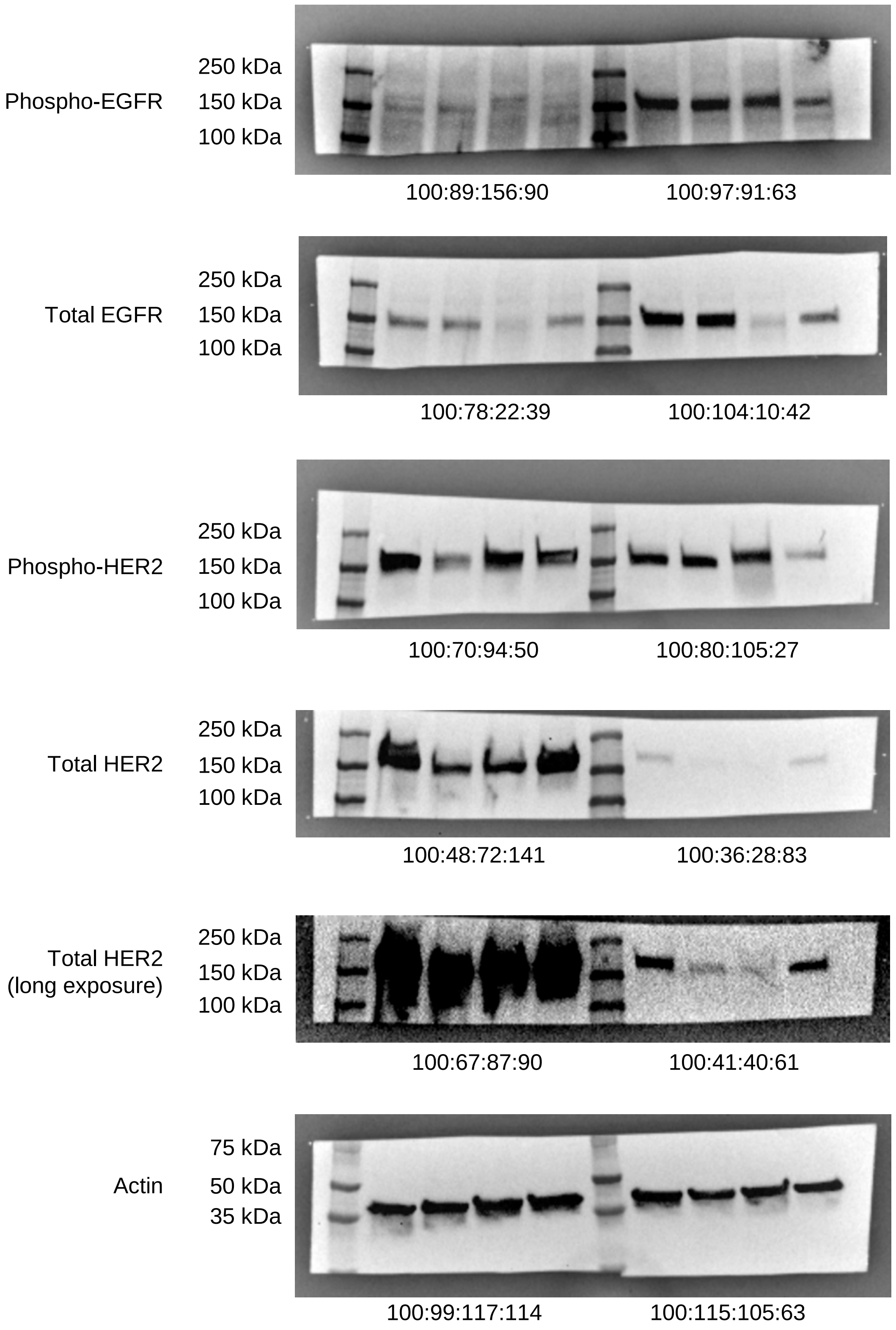
Phospho-EGFR
250 kDa
150 kDa
100 kDa
100:89:156:90
100:97:91:63
Total EGFR
250 kDa
150 kDa
100 kDa
100:78:22:39
100:104:10:42
Phospho-HER2
250 kDa
150 kDa
100 kDa
100:70:94:50
100:80:105:27
Total HER2
250 kDa
150 kDa
100 kDa
100:48:72:141
100:36:28:83
Total HER2
(long exposure)
250 kDa
150 kDa
100 kDa
100:67:87:90
100:41:40:61
Actin
75 kDa
50 kDa
35 kDa
100:99:117:114
100:115:105:63

Figure S4

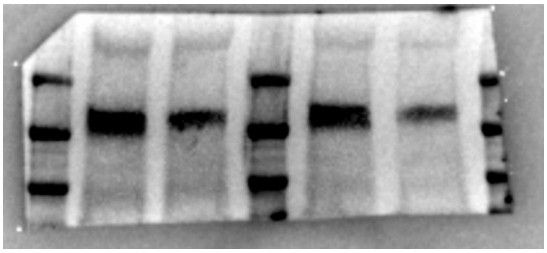

250 kDa
150 kDa Phospho-HER2
100 kDa

100:45

Total HER2
250 kDa
150 kDa
100 kDa

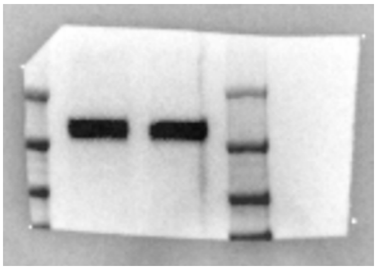

100:101

Actin
75 kDa
50 kDa
35 kDa

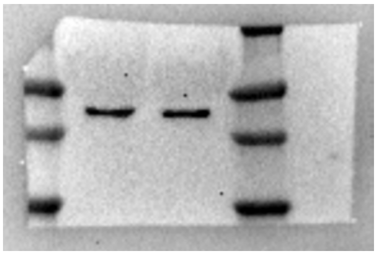

100:107

Figure S5

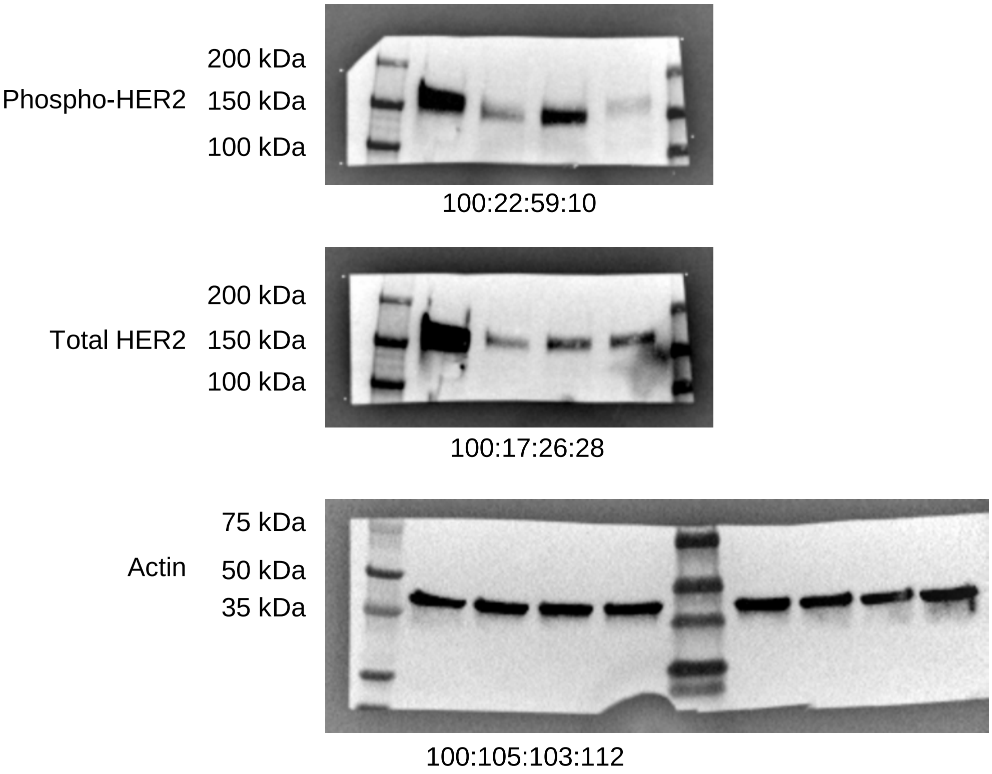
Phospho-HER2
200 kDa
150 kDa
100 kDa
100:22:59:10
Total HER2
200 kDa
150 kDa
100 kDa
100:17:26:28
Actin
75 kDa
50 kDa
35 kDa
100:105:103:112

Figure S6

Phospho-EGFR

250 kDa
150 kDa
100 kDa

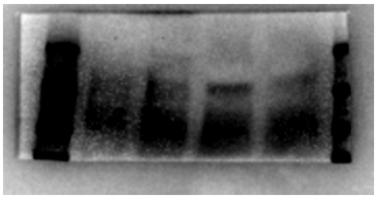

100:111:155

Actin

75 kDa
50 kDa
35 kDa

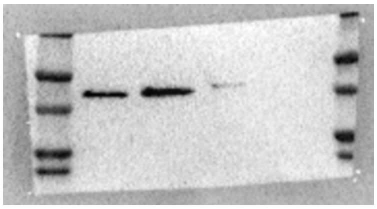

100:152:16

Total EGFR

250 kDa
150 kDa
100 kDa

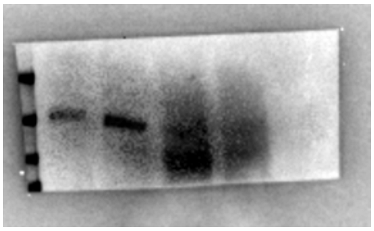

100:181:141

Actin

75 kDa
50 kDa
35 kDa

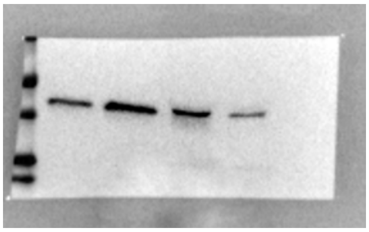

100:187:141

Figure S7

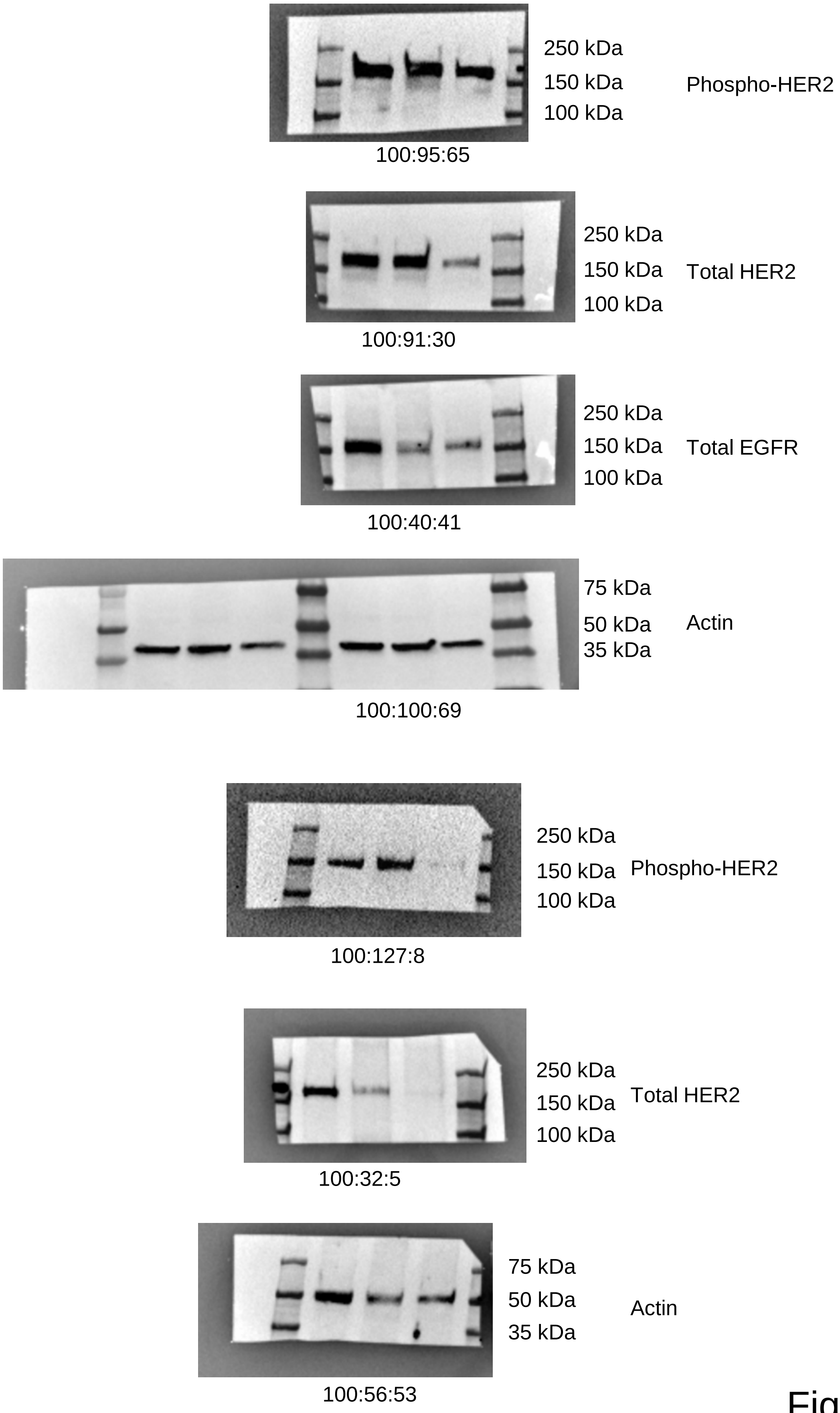
250 kDa
150 kDa
100 kDa
Phospho-HER2
100:95:65
250 kDa
150 kDa
100 kDa
Total HER2
100:91:30
250 kDa
150 kDa
100 kDa
Total EGFR
100:40:41
75 kDa
50 kDa
35 kDa
Actin
100:100:69
250 kDa
150 kDa
100 kDa
Phospho-HER2
100:127:8
250 kDa
150 kDa
100 kDa
Total HER2
100:32:5
75 kDa
50 kDa
35 kDa
Actin
100:56:53

Figure S8

Phospho-EGFR

200 kDa
150 kDa
100 kDa

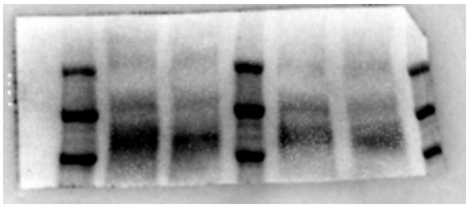

100:63

Actin

75 kDa
50 kDa
35 kDa

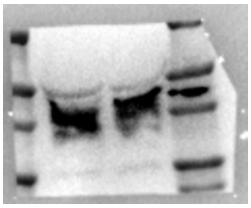

100:96

Figure S9

HER2

250 kDa
150 kDa
100 kDa

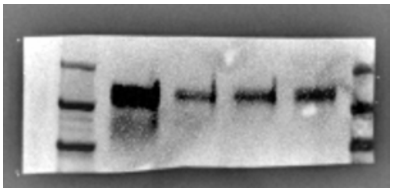

100:35:36:33

Actin

75 kDa
50 kDa
35 kDa

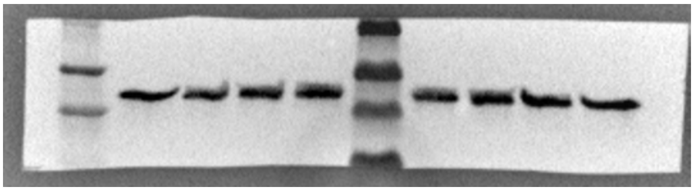

100:84:95:124

250 kDa
150 kDa
100 kDa

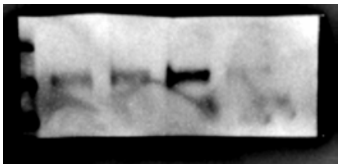

EGFR

100:103:192:53

75 kDa
50 kDa
35 kDa

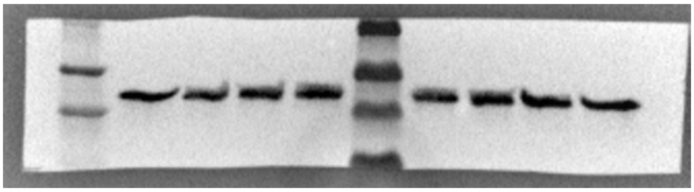

Actin

100:126:127:104

250 kDa
150 kDa
100 kDa

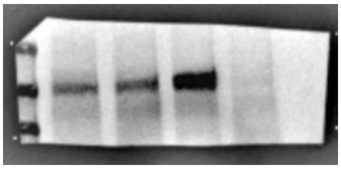

Phospho-EGFR

100:125:254:24

250 kDa
150 kDa
100 kDa

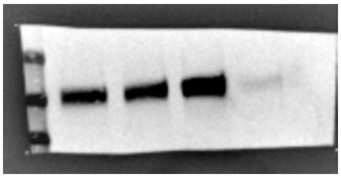

EGFR

100:100:124:16

75 kDa
50 kDa
35 kDa

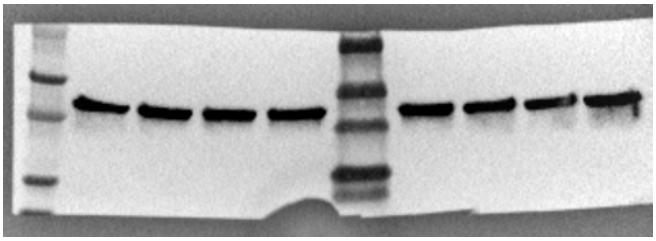

Actin

100:92:77:121

Figure S10
